# Roles of XBP1s in Transcriptional Regulation of Target Genes

**DOI:** 10.3390/biomedicines9070791

**Published:** 2021-07-08

**Authors:** Sung-Min Park, Tae-Il Kang, Jae-Seon So

**Affiliations:** Department of Medical Biotechnology, Dongguk University, Gyeongju 38066, Gyeongbuk, Korea; psmin06@naver.com (S.-M.P.); kti3935@naver.com (T.-I.K.)

**Keywords:** XBP1s, IRE1, ATF6, ER stress, unfolded protein response, UPR, RIDD

## Abstract

The spliced form of X-box binding protein 1 (XBP1s) is an active transcription factor that plays a vital role in the unfolded protein response (UPR). Under endoplasmic reticulum (ER) stress, unspliced *Xbp1* mRNA is cleaved by the activated stress sensor IRE1α and converted to the mature form encoding spliced XBP1 (XBP1s). Translated XBP1s migrates to the nucleus and regulates the transcriptional programs of UPR target genes encoding ER molecular chaperones, folding enzymes, and ER-associated protein degradation (ERAD) components to decrease ER stress. Moreover, studies have shown that XBP1s regulates the transcription of diverse genes that are involved in lipid and glucose metabolism and immune responses. Therefore, XBP1s has been considered an important therapeutic target in studying various diseases, including cancer, diabetes, and autoimmune and inflammatory diseases. XBP1s is involved in several unique mechanisms to regulate the transcription of different target genes by interacting with other proteins to modulate their activity. Although recent studies discovered numerous target genes of XBP1s via genome-wide analyses, how XBP1s regulates their transcription remains unclear. This review discusses the roles of XBP1s in target genes transcriptional regulation. More in-depth knowledge of XBP1s target genes and transcriptional regulatory mechanisms in the future will help develop new therapeutic targets for each disease.

## 1. Introduction

The endoplasmic reticulum (ER) plays an essential role in the synthesis, folding, assembly, and modification of transmembrane or secretory proteins [1,2]. Moreover, this intracellular organelle participates in calcium storage, lipid synthesis, and the detoxification of xenobiotics, drugs, and metabolic by-products [1,2,3]. Proteins are modified and oligomerized in the ER lumen; correctly folded proteins can thus exit the ER and reach their final destination [4]. However, the perturbation of ER homeostasis results in the accumulation of misfolded or unfolded protein in the ER lumen, leading to ER stress [5,6]. ER stress is closely related to various environmental, physiological, and pathological disturbances, such as ER calcium deficiency, hypoxia, oxidative stress, malnutrition, infectious pathogens, cancers, neurodegenerative disorders, and metabolic diseases [6,7,8].

**Figure 1:**
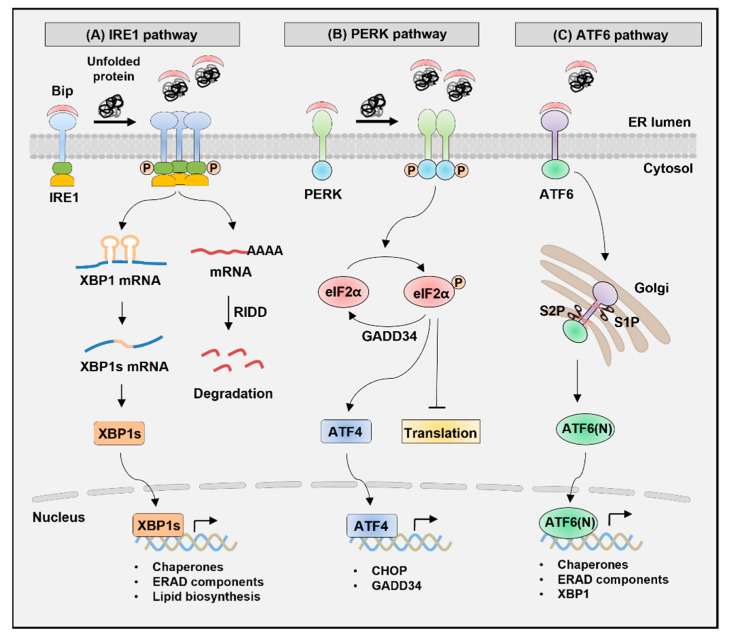
Signaling pathways of UPR. Signaling pathways of UPR are mediated by three ER-resident proteins: IRE1, PERK, and ATF6. In the absence of ER stress, BiP binds to the ER luminal domain of the sensor proteins and inhibits their activation. However, ER stress induces the dissociation of BiP from the sensors, which activates UPR transducers. (**A**) IRE1 pathway: IRE1 has serine/threonine kinase and RNase domains in the cytoplasmic region. Upon ER stress, IRE1 is activated via oligomerization and autophosphorylation, leading to increased RNase activity. Activated IRE1 recognizes the stem-loop structure of *Xbp1* mRNA and induces unconventional splicing by cleaving 26 intronic nucleotides. The spliced *Xbp1* mRNA is translated by frameshift into an active transcription factor, XBP1s. Then, XBP1s upregulates the expression of UPR target genes, including ER chaperones, ERAD components, and lipid biosynthetic enzymes. Furthermore, IRE1 recognizes various mRNAs besides *Xbp1* mRNA as substrates under ER stress and induces the degradation of these mRNAs, a process known as regulated IRE-dependent decay (RIDD). RIDD is a mechanism that resolves ER stress by degrading mRNAs encoding ER-targeted proteins, thereby reducing protein loads into ER. (**B**) PERK pathway: PERK has a serine/threonine kinase domain in its cytoplasmic region. ER stress induces PERK activation via oligomerization and autophosphorylation in the kinase domain. Activated PERK then phosphorylates the serine 51 residue of eIF2α, resulting in alleviation of ER stress by attenuating translation. By contrast, phosphorylated eIF2α selectively promotes the translation of ATF4, which activates the transcription of CHOP and GADD34. After ER stress resolution, GADD34 interacts with PP1 to induce eIF2α dephosphorylation, restoring protein translation. However, if ER stress is not resolved, CHOP induces apoptosis. (**C**) ATF6 pathway: ATF6 has an N-terminal b-ZIP domain in the cytoplasmic region. ER stress induces ATF6 translocation from ER to the Golgi apparatus, where ATF6 is cleaved by S1P and S2P. This proteolytic cleavage produces the N-terminal region of ATF6, referred to as ATF6(N). ATF6(N) functions as an active transcription factor and upregulates target genes encoding ER chaperones, ERAD components, and XBP1. IRE1, inositol-requiring enzyme 1; PERK, protein kinase R-like ER kinase; ATF6, activating transcription factor 6; RNase, endoribonuclease; XBP1, X-box binding protein 1; ERAD, ER-associated protein degradation; eIF2α, eukaryotic translation initiation factor 2α; ATF4, activating transcription factor 4; CHOP, C/EBP homologous protein; GADD34, growth arrest and DNA damage-inducible protein 34; PP1, protein phosphatase 1; S1P, site-1 protease.

In response to ER stress, the protein quality control system of ER activates three distinct signaling pathways known as the unfolded protein response (UPR) to restore ER homeostasis (Figure 1) [9]. ER stress is recognized by the following ER sensor proteins in mammalian cells: inositol-requiring enzyme 1 (IRE1), protein kinase R (PKR)-like ER kinase (PERK), and activating transcription factor 6 (ATF6) [9]. Each of these transmembrane proteins controls distinct branches of UPR that regulate unique transcriptional or translational programs [10,11]. UPR alleviates ER stress primarily via three mechanisms [9,10,11,12]: (1) UPR increases the protein folding capacity of ER by inducing the transcription of various genes encoding molecular chaperones and folding enzymes; (2) UPR attenuates protein translation, thereby reducing the burden on ER by inhibiting the translocation of new proteins into ER; and (3) misfolded proteins in ER are retrotransported to the cytosol, polyubiquitinated, and degraded by the 26S proteasome, a process called ER-associated protein degradation (ERAD) [13,14]. However, if ER stress is not relieved or is too severe, UPR induces apoptosis to remove the damaged cells [15,16,17].

Among the UPR transducers, IRE1 is the most evolutionarily conserved protein from yeast to mammals. IRE1 has kinase and endoribonuclease (RNase) activities, and activated IRE1 induces *Xbp1* mRNA into its mature form via unconventional splicing [18,19]. The spliced *Xbp1* mRNA is translated into the potent transcription factor XBP1s, thereby promoting the transcription of UPR-related genes encoding ER chaperones and folding enzymes. This process increases the ER folding capacity. Moreover, XBP1s regulates specific gene transcription, depending on the cell type, to control diverse cellular functions, including lipid metabolism, glucose biosynthesis, and immune responses [20,21,22,23,24]. Many recent reviews cover various physiological characteristics of ER stress and describe the contribution of ER stress to the pathogenesis of diseases (such as cancer, diabetes, and neurodegenerative, metabolic, and inflammatory disorders) [25,26,27,28,29]. This review describes the possible mechanisms underlying the XBP1s-mediated transcriptional regulation of target genes. We discuss the roles of XBP1s, including the transcription of target genes, XBP1s-interacting proteins, and regulation of XBP1s activity in various types of cells.

## 2. Signaling Pathways of UPR

UPR is a signal transduction pathway induced in response to ER stress, which is initiated by recognizing unfolded or misfolded proteins in the ER lumen (Figure 1) [24]. The three UPR transducers, IRE1, PERK, and ATF6, are transmembrane proteins with luminal and cytosolic domains. In unstressed cells, the ER chaperone-binding immunoglobulin protein (BiP, also known as GRP78) binds to the luminal domain of the sensor proteins, inhibiting their activity [30]. However, the accumulation of misfolded or unfolded protein promotes the dissociation of BiP from the luminal domain of transducers, leading to the oligomerization of IRE1 and PERK and phosphorylation in their kinase domain [31]. As a result, subsequent downstream signaling pathways are induced, resulting in the activation of the effector function of each UPR branch. The dissociation of BiP from the ATF6 luminal domain induces the translocation of ATF6 to the Golgi apparatus where it undergoes proteolytic cleavage to release the N-terminal domain, which acts as a transcription factor to initiate UPR responses [32].

### 2.1. IRE1 Pathway

IRE1, an ER-resident type-I transmembrane protein, is the most evolutionarily conserved protein in the UPR pathways [33]. Mammalian cells express the two isoforms IRE1α and IRE1β, which have the serine/threonine kinase and endoribonuclease (RNase) domains in their cytoplasmic region [34]. Upon ER stress, IRE1α is activated via oligomerization and autophosphorylation, leading to increased RNase activity [35]. Then, the activated IRE1α recognizes the stem-loop structure of *Xbp1* mRNA containing a consensus sequence (5′-CUGCAG-3′) and induces unconventional splicing by cleaving 26 intronic nucleotides (531–556) [18,36]. *Xbp1* mRNA usually encodes an unstable protein XBP1u (unspliced form XBP1, 267 amino acids in mice), whereas spliced mRNA is translated into XBP1s (371 amino acids) by a frameshift [19]. XBP1s is an active transcription factor with a basic leucine zipper (b-ZIP) domain in the new C-terminus that upregulates the expression of UPR target genes, including ER chaperones (*Dnajb9*, *Dnajb11*, *Pdia3*, and *Dnajc3*), ERAD components (*Edem1*, *Herpud1*, and *Hrd1*), folding enzyme (*Pdia6*), and ER translocon (*Sec61a1*) [23,37,38,39]. Moreover, XBP1s contributes to a wide range of biological processes by regulating gene expression as per cell type [40,41,42,43,44,45].

### 2.2. PERK Pathway

PERK, a type-I transmembrane protein, has a serine/threonine kinase domain in its cytoplasmic region [9]. Activated PERK phosphorylates the serine 51 residue of the eukaryotic translation initiation factor 2α (eIF2α) [46]. eIF2α phosphorylation then alleviates ER stress by translational attenuation, which reduces the amount of protein entering ER. By contrast, phosphorylated eIF2α selectively promotes the translation of activating transcription factor 4 (*Atf4*) mRNA. In unstressed cells, ATF4 translation is suppressed by inhibitory upstream open reading frames (uORF) located in the 5′-untranslated region (UTR). However, eIF2α phosphorylation induces a ribosome bypass of the inhibitory uORF, leading to increased ATF4 translation [47,48,49]. Increased ATF4 activates the transcription of C/EBP homologous protein (*Chop*) and growth arrest and DNA damage-inducible protein 34 (*Gadd34*). When ER stress is not resolved, CHOP induces apoptosis by increasing the transcription of proapoptotic proteins, such as death receptor 5 (DR5) and BCL-2 interacting mediator of cell death (BIM) [50,51,52]. However, after ER stress resolution, GADD34 interacts with protein phosphatase 1 (PP1) to induce eIF2α dephosphorylation, restoring protein translation [53,54].

### 2.3. ATF6 Pathway

In mammals, ATF6 has two homologous proteins, ATF6α and ATF6β. ATF6 is an ER-resident type-II transmembrane protein with a cytoplasmic N-terminal b-ZIP domain [55]. ER stress induces ATF6 translocation from ER into the Golgi apparatus where ATF6 is cleaved by site-1 protease (S1P) and site-2 protease (S2P) to release the cytoplasmic region [56,57]. Consequently, the 50-kDa fragment of the N-terminal region, referred to as ATF6(N), migrates to the nucleus and functions as an active transcription factor [55,56]. ATF6 binds to the ER stress response element (ERSE), a *cis*-acting element, in the promoter of target genes, such as ER chaperones (*Hspa5*, *Hsp90b1*, and *Calr*), *Ddit3*, and *Xbp1* [58,59,60]. ERSE has the consensus sequence of CCAAT-N_9_-CCACG and is necessary to induce major ER chaperones to recover ER homeostasis [58,60]. In addition, ATF6 binds to ERSE-II with the consensus sequence of ATTGG-N-CCACG located in the promoter region of *Herpud1* [61]. ATF6(N) binds to the CCACG of ERSE only when CCAAT is bound by the general transcription factor NF-Y/CBF [59,62].

## 3. Transcriptional Regulation of Target Genes by XBP1s

**Figure 2:**
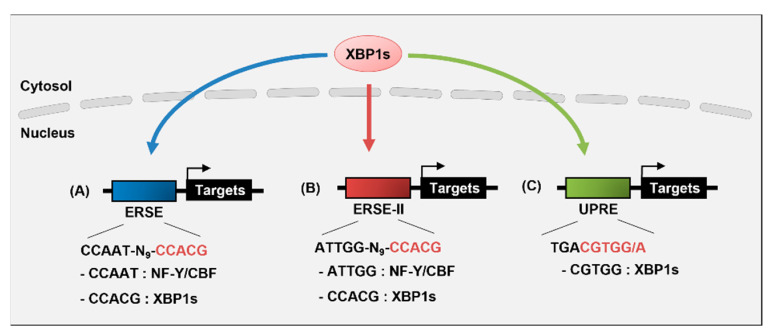
*Cis*-acting elements of XBP1s. XBP1s is an active transcription factor and plays an important role in the transcriptional regulation of UPR target genes encoding ER chaperones, folding enzymes, and ERAD components. XBP1s binds to three *cis*-acting elements: ERSE, ERSE-II, and UPRE. (**A**) ERSE has the consensus sequence of CCAAT-N_9_-CCACG and is located in the promoter of several genes, such as ER chaperones (*Hspa5*, *Hsp90b1*, and *Calr*), *Ddit3*, and *Xbp1*. (**B**) ERSE-II has the consensus sequence of ATTGG-N-CCACG and is located in the promoter of *Herpud1*. The CCACG of ERSE and ERSE-II is the binding site for XBP1s or ATF6, and ATTGG is the binding site for NF-Y/CBF. (**C**) UPRE has the consensus sequence of TGACGTGG/A and XBP1s preferentially binds to CGTGG of UPRE.

XBP1 is a member of the cAMP-response element-binding (CREB)/activating transcription factor (ATF) b-ZIP family of transcription factors [63]. Activated IRE1 processes *Xbp1* mRNA into a mature form via unconventional splicing, enabling active transcription factor XBP1s expression [64]. XBP1s translocates to the nucleus and induces the transcription of UPR-related genes by binding to specific *cis*-acting elements, such as ERSE, ERSE-II, and unfolded protein response element (UPRE), in mammals (Figure 2) [39,65,66]. The CCACG section of ERSE or ERSE-II provides specificity for the binding of XBP1s and ATF6, whereas UPRE is preferentially bound by XBP1s [38,65]. UPRE contains the consensus sequence of TGACGTGG/A, and the sequence complementary to the underlined sequence is consistent with the CCACG of ERSE for XBP1s binding [66]. ATF6 binds to ERSE or ERSE-II in an NF-Y-dependent manner, whereas XBP1s binds to these elements independently [59,65,66]. Under severe ER stress, XBP1s upregulates the transcription of the Krüppel-like factor 9 (*KLF9*) by binding to the UPRE of its promoter [67]. KLF9, in turn, promotes Ca^2+^ release from ER and cell death by increasing the transcription of the ER calcium storage regulator transmembrane protein 38B (*TMEM38B*) and inositol 1,4,5-trisphosphate receptor type 1 (*ITPR1*).

**Figure 3:**
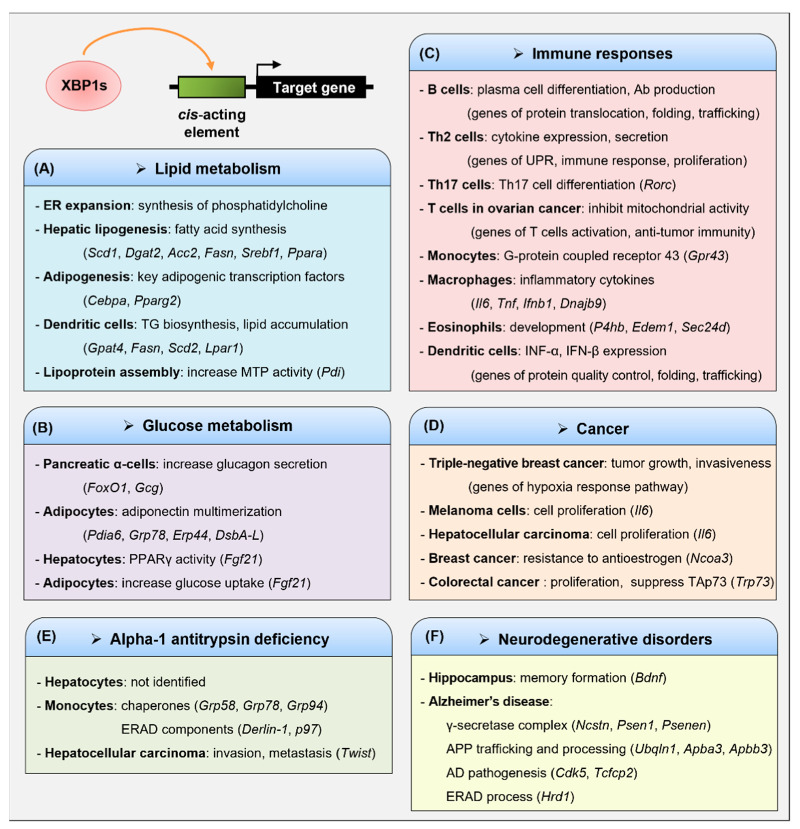
Transcriptional regulation of target genes by XBP1s. XBP1s regulates the transcription of various target genes that constitute unique subsets depending on the specific stimuli and cell types. In many studies, XBP1s has been shown to regulate genes that are involved in various cellular processes, such as the ER stress response, protein secretion, lipid and glucose metabolism, immune responses, and cancer development. XBP1s was initially known to play an important role in the development and maintenance of highly secretory cells, such as plasma cells, pancreatic acinar cells and β-cells, hepatocytes, and intestinal Paneth cells. (**A**) Moreover, XBP1s is a crucial transcription factor for lipid metabolisms involved in the biosynthesis of ER membrane, hepatic lipogenesis, and adipocyte differentiation. (**B**) XBP1s plays an important role in glucose metabolism by regulating the transcription of UPR- and non-UPR-associated genes in hepatocytes, pancreatic cells, and adipocytes. (**C**) Several studies have reported that XBP1s regulates the development, differentiation, and immune responses of various immune cells, such as B cells, T cells, macrophages, and dendritic cells. (**D**) In addition, XBP1s is a crucial regulator of tumorigenesis by regulating the transcription of tumor cell-related genes. XBP1s regulates the development, progression, and metastasis of various tumors, such as triple-negative breast cancer (TNBC), melanoma, hepatocellular carcinoma (HCC), ER-positive breast cancer, and colorectal cancer. (**E**) Alpha-1 antitrypsin (AAT) is mainly produced by hepatocytes, whereas Z variant of AAT (ZAAT) induces the accumulation of misfolded AAT, leading to AAT deficiency. The function and target genes of XBP1s in AATD have not been well elucidated. (**F**) XBP1s plays an important role in neurodegenerative disorders and brain function.

XBP1s-regulated genes are diverse and constitute unique subsets depending on the specific stimuli and cell types (Figure 3). Studies have shown that XBP1s regulates genes that are involved in diverse cellular processes, such as the ER stress response, secretory function, lipid metabolism, glucose homeostasis, and the inflammatory response [26,37,42,68]. Thus, XBP1s plays an important role in developing and maintaining highly secretory cells, such as plasma cells, pancreatic acinar cells and β-cells, hepatocytes, and intestinal Paneth cells [43,44,45,69,70]. Deleting XBP1 increases susceptibility to intestinal inflammation or causes insulin resistance and type 2 diabetes [45,71]. Moreover, XBP1s enhances the development and survival of dendritic cells (DC) and proinflammatory cytokine production in macrophages [72,73]. These results indicate an important role for XBP1s in various physiological and pathological processes.

At present, investigation into XBP1s is limited to evaluating its ability to regulate cell function and disease. Although recent studies have identified numerous potential target genes for XBP1s in various cells, the mechanism by which XBP1s regulates the transcription of each gene is not fully understood. Information on the genes and cellular functions that XBP1s regulates in each cell may help analyze the mechanisms of transcriptional regulation by XBP1s at the molecular level.

### 3.1. Lipid Metabolism

XBP1s is a crucial transcription factor as a mediator of the ER stress response and plays a crucial role in the lipid biosynthesis of the ER membrane, hepatic lipogenesis, and adipocyte differentiation [21,74,75,76]. XBP1s increases ER expansion by inducing the synthesis of phosphatidylcholine (PtdCho), the primary phospholipid of the ER membrane [74]. It also increases the activity of choline-phosphate cytidylyltransferase (CCT) and choline phosphotransferase 1 (CPT1) enzymes, which participate in the cytidine diphosphocholine pathway for PtdCho biosynthesis; however, XBP1s does not alter their transcript levels. Although not elucidated in that study, XBP1s might regulate certain target genes, which increase the activity of CCT and CPT1 enzymes.

XBP1s increases hepatic lipogenesis by regulating gene expression involved in fatty acid synthesis [21]. XBP1s is induced by a high carbohydrate diet in the liver and subsequently upregulates the expression of lipogenic genes, such as stearoyl-CoA desaturase 1 (*Scd1*), diacylglycerol O-acyltransferase 2 (*Dgat2*), and acetyl-CoA carboxylase 2 *(Acc2*) by binding to their promoter regions. Therefore, deleting XBP1 in the liver causes hypocholesterolemia and hypotriglyceridemia. This XBP1s-mediated transcriptional regulation is independent of the carbohydrate response element-binding protein (ChREBP) and sterol regulatory element-binding protein (SREBP) that are needed for lipid synthesis. Of note, the UPR target genes *Edem*, *Dnajb9*, and *Sec61a1*, were only modestly decreased in the XBP1-deficient liver. Moreover, XBP1s enhances insulin-mediated hepatic lipogenesis [75]. A high-fat diet induces hyperinsulinemia and insulin resistance in mice and elevates XBP1s expression in the liver. In addition, chronic exposure to insulin induces XBP1s expression and initiates a lipogenic program in the liver. This is because XBP1s stimulates the promoter activity of fatty acid synthase (*Fasn*) and *Srebf1* (encoding SREBP-1c) genes. Additionally, insulin activates the binding of XBP1s to the promoter of key lipogenic gene *Srebf1*, which contains the CCACG sequence (the XBP1 binding site of ERSE). Prolonged fasting or ketogenic diet activates the IRE1α–XBP1 signaling pathway in the liver [77]. Hence, increased XBP1s binds to the UPRE-like region of the peroxisome proliferator-activated receptor α (*Ppara*) promoter and upregulates the expression of PPARα, a master regulator of starvation responses, to promote fatty acid β-oxidation and ketogenesis.

Several studies have shown that XBP1s plays a crucial role in adipocyte differentiation by regulating morphological and functional transformation during adipogenesis [76]. XBP1s directly regulates the transcription of CCAAT/enhancer-binding protein α (*Cebpa*), which encodes a key adipogenic transcription factor C/EBPα, by binding to a proximal promoter containing the CCACG sequence. Thus, XBP1 deficiency reduces the mRNA level of *Cebpa*, attenuating adipocyte differentiation. XBP1s also regulates adipogenesis by increasing PPARγ2, the master regulator of adipogenesis [78]. Moreover, XBP1s enhances the activity of the *Pparg* promoter and directly binds to the CCACG motif in adipocytes. The importance of XBP1s in lipid biosynthesis has been demonstrated in the study of DCs [79]. Tumor-associated DCs (tDCs) from ovarian cancer (OvCa) exhibit increased XBP1s, which induces triglyceride (TG) biosynthesis and abnormal lipid accumulation. This reduces the anti-tumor immunity of DCs. Transcriptional profiling identified the downregulation of several known XBP1s and UPR target genes in XBP1-deficient tDCs. By contrast, OvCa tDCs show a marked increase in TG biosynthetic gene expression, including *Gpat4*, *Fasn*, *Scd2*, and *Lpar1*. It is therefore important to find a common or specific transcriptional mechanism by comparing the location and nucleotide sequences of the XBP1s binding sites for genes involved in lipid metabolism.

Hepatocytes secrete apolipoprotein B (ApoB)-containing lipoprotein particles (very-low-density lipoprotein (VLDL) and low-density lipoprotein (LDL)) to transport cholesterol and fatty acids to peripheral tissues [80]. XBP1s increases the assembly and secretion of hepatic VLDL by inducing protein disulfide isomerase (PDI) [81]. A hepatocyte-specific deletion of IRE1α inhibits the assembly of TG-rich VLDL but does not affect the synthesis or secretion of ApoB, de novo lipogenesis, and TG synthesis [81,82]. IRE1α deletion downregulates PDI expression, thereby reducing the activity of microsomal triglyceride-transfer protein (MTP) required for lipoprotein assembly [81,83,84]. Thus, IRE1α deletion in hepatocytes results in hepatic steatosis owing to increased TG accumulation caused by defects in VLDL assembly [82]. Taken together, the above results indicate that XBP1s is an important regulator for lipogenesis-related genes transcription and a potential therapeutic target for dyslipidemia.

### 3.2. Glucose Metabolism

XBP1s plays an important role in glucose metabolism by regulating UPR- and non-UPR-associated gene transcription in hepatocytes, pancreatic cells, and adipocytes [42,85,86,87]. XBP1 deficiency in pancreatic α-cells induces glucose intolerance and insulin resistance caused by the dysregulation of glucagon secretion [85]. Of note, XBP1 knockdown in α-cells impairs insulin signaling by increasing the phosphorylation of both IRE1α and c-Jun N-terminal kinase (JNK). Moreover, XBP1 deficiency decreases the expression of the *Hspa5* and glucagon (*Gcg*) genes because the nuclear amount of FoxO1, which is known to bind to the promoter of the preproglucagon gene, is reduced. These results suggest the possibility that XBP1s directly regulate *Foxo1* or *Gcg* transcription. XBP1s regulates systemic glucose homeostasis by promoting adiponectin multimerization in adipocytes [86]. Adiponectin is an insulin-sensitizing hormone, and adiponectin levels are inversely correlated with glucose intolerance and type 2 diabetes [88]. Global transcriptional profiling revealed that XBP1s increases the expression of several ER chaperones involved in adiponectin maturation, including PDI-associated 6 (PDIA6), glucose-regulated protein 78 (GRP78), ER protein 44 (ERP44), and disulfide bond oxidoreductase A-like protein (DsbA-L). XBP1s directly binds to the promoter of these genes and increases the promoter activity. Therefore, XBP1s elevates the serum levels of high-molecular-weight adiponectin, resulting in improved glucose tolerance and insulin sensitivity.

XBP1s enhances insulin-stimulated glucose uptake by increasing PPARγ activity in adipocytes [87]. Fibroblast growth factor 21 (FGF21) functions as a PPARγ-activating protein induced by XBP1s in hepatocytes [89]. In insulin-treated adipocytes, XBP1s also binds to the promoter region of *Fgf21*, and increases FGF21 expression, thereby resulting in the upregulation of PPARγ activity [87]. Therefore, XBP1s reduces palmitate-induced insulin resistance by increasing insulin signaling and adiponectin secretion and inhibiting pro-inflammatory adipokine secretion.

### 3.3. Immune Responses

ER stress modulates innate and adaptive immunity and has been shown to be closely associated with various immune disorders, including diabetes, rheumatoid arthritis, atherosclerosis, myositis, and inflammatory bowel disease [26,45,90,91,92,93,94]. Several studies have reported that XBP1s plays a crucial role in the development, differentiation, and immune responses of various immune cells [12,23]. In immune cells, the role of XBP1s was first shown as an essential transcription factor for the differentiation of mature B cells to plasma cells [43].

#### 3.3.1. B Cells

XBP1s plays a vital role in plasma cell differentiation and immunoglobulin (Ig) production [43,95,96]. XBP1s expression increases as B cells differentiate into plasma cells, which initiates before the increased synthesis of nascent Ig heavy and light chains during the differentiation of antibody-secreting B cells [97]. XBP1-deficient lymphoid chimeric mice show a severe defect in the generation of plasma cells and Ig secretion. However, the number of B lymphocytes is normal, suggesting that XBP1s is required for B lymphocytes’ terminal differentiation to plasma cells [43]. BLIMP-1 is a major transcription factor that induces the differentiation of B cells into plasma cells, and BLIMP-1 levels are normal in XBP1-deficient B cells [95]. By contrast, BLIMP-1-deficient B cells fail to express XBP1, indicating that XBP1 is at the downstream of BLIMP-1. The above results show that the failure of XBP1^−/−^ B cells to differentiate into plasma cells is not caused by BLIMP-1 reduction [95]. Gene expression profiling demonstrated that XBP1 deficiency fails to upregulate many genes that encode secretory pathway components. This group also investigated XBP1s target genes by comparing gene expression between the XBP1s- and XBP1u-transduced cells. XBP1s-induced genes encode proteins involved in the translocation of protein across the ER membrane (SRP54, SRPR, SSR3, SSR4, RPN1, TRAM1, and SPC22/23), ER protein folding (ERP70, PPIB, GRP58, FKBP11, ERdj4, and GRP78), protein glycosylation (GCS1, DDOST, and DAD1), and vesicle trafficking (SEC23B, SEC24C, OS-9, GOLGB1, and MCFD2). Hence, XBP1s is an important factor to regulate secreting apparatus, protein synthesis, and ER expansion in B cells. Although many XBP1s target genes have been identified in B cells, their transcriptional regulatory mechanisms, including cis-acting elements, are still unknown.

#### 3.3.2. T Cells

CD4^+^ T cells differentiate into several helper T (Th) lineages, including T helper type 1 (Th1), Th2, Th9, Th17, follicular helper T cells (Tfh), and regulatory T cells (Tregs), depending on the microenvironment [98,99]. Classification of the Th subtype is based on the production of specific cytokines and transcription factors that are important for differentiation [99]. XBP1s regulates genes that control diverse physiological aspects of Th2 cells [100]. IRE1α–XBP1s signaling controls cytokine expression, secretion, and cell proliferation in Th2 cells. Th2 cells are characterized by the secretion of interleukin (IL)-4, IL-5, IL-10, and IL-13 and are involved in allergy, asthma, and helminth infection [101]. The differentiation of naive T cells into Th2 cells increases XBP1s expression by inducing IRE1α expression and phosphorylation [100]. An IRE1α RNase inhibitor, 4μ8c, was used to identify XBP1s-regulated target genes and predict their biological roles. A genome-wide transcriptomic analysis of 4μ8c-treated and untreated Th2 cells showed that differentially expressed genes are associated with UPR, signal transduction, cytokine production, proliferation, the cell cycle, developmental processes, and the immune response [100]. Chromatin immunoprecipitation sequencing (ChIP-seq) analysis identified XBP1s-binding sites, most of which are located within the promoter (33%), intron (36%), and intergenic regions (27%). Enriched DNA motifs of those regions revealed the XBP1s-binding motif contained the consensus sequence CACGT and NF-Y-binding motif. XBP1s target genes and their transcriptional regulatory networks are related to ER stress, the immune response, the cell cycle, and proliferation. The 4μ8c inhibits the expression of Th2 cytokines, including IL-5 and IL-13, and cell proliferation in Th2 cells.

Th17 cells express IL-17 and the lineage-specific transcription factor RORγt and are associated with inflammatory and autoimmune disorders [102]. A recent study demonstrated that cellular stress, such as hypoxia, hypoglycemia, and isotonic stress, increases the generation of Th17 cells via XBP1s activity even in the absence of TGF-β [103]. ER stress inducers, tunicamycin, thapsigargin, and CPA, also enhance Th17 cell polarization; however, the treatment of stress inhibitor or XBP1 deficiency in naive T cells reduces Th17 cell differentiation and expression of IL-17 and RORγt. These results indicate that XBP1s may directly regulate the transcription of *Rorc*, which encodes RORγt.

XBP1s inhibits the antitumor immunity of T cells in OvCa [104]. OvCa microenvironments inhibit glucose uptake in T cells, leading to defects in N-linked glycosylation. These disturbances induce ER stress and increase XBP1s in T cells, thereby inhibiting mitochondrial activity and interferon (IFN)-γ production. Analysis using patient samples showed that XBP1s upregulation is associated with the decreased infiltration of T cells into tumors and reduced production of IFN-γ. XBP1s also decreases the influx of glutamine required for mitochondrial respiration by inhibiting the abundance of glutamine carriers. Hence, XBP1 deficiency in T cells restores anti-tumor capacity and delays malignant progression, resulting in the increased survival of mice with OvCa. The transcriptional profiling of wild-type versus XBP1-deficient CD4^+^ T cells isolated from the peritoneal cavity of mice with OvCa identified 151 differentially expressed genes. Upregulated genes in XBP1-deficient CD4^+^ T cells are related to T-cell activation (*Cd69*, *Cd44*, *Cd28*, and *Nfkb1*) and mediators of antitumor immunity (*Ccl5*, *Ifng*, *Klrk1*, and *Fasl*). Thus, XBP1-deficient CD4^+^ T cells increase IFN-γ production and antitumor immunity. Although transcriptomic analysis has shown that the expression of many genes is affected by XBP1 deficiency, it is still unknown which genes are XBP1s targets. Further studies are required to locate XBP1s target genes and analyze the transcriptional mechanisms regulated by XBP1s.

#### 3.3.3. Monocytes and Macrophages

XBP1s upregulates the expression of G-protein-coupled receptor 43 (GPR43) in human monocytes [105]. GPR43 recognizes short-chain fatty acids and plays a crucial role in preventing obesity, colitis, asthma, and arthritis [106,107]. GPR43 is highly expressed in monocytes, and lipopolysaccharide (LPS) or granulocyte-macrophage colony-stimulating factor (GM-CSF) increases the transcript levels of *Gpr43* [108]. XBP1s binds to the core promoter region (−58 to −33) of *Gpr43* and enhances its transcription [105]. Mutation in the XBP1s binding sites alters *Gpr43* promoter activity to basal levels. XBP1 knockdown reduces GPR43 expression, whereas tumor necrosis factor α (TNF-α) upregulates GPR43 expression by activating XBP1s.

Macrophages that differentiate from monocytes are essential for primary defense and innate immunity against pathogens [109]. Toll-like receptor 2 (TLR2) or TLR4 activates XBP1s in macrophages; in turn, XBP1s promotes the production of inflammatory cytokines, including IL-6, TNF, and IFN-β [73]. Thus, XBP1 deficiency increases bacterial infection in mice by reducing the levels of these cytokines. ChIP experiments showed the recruitment of XBP1s to the promoters of *Il6* and *Tnf*. LPS synergistically increases the expression of IFN-β in macrophages with activated UPR [110,111]. This increase in IFN-β is dependent on XBP1s expression [110]. XBP1s binds to a conserved site, located 6.1 kb downstream of the *Ifnb1* gene, and interacts with IRF-3 and CBP/p300 [111]. Sequence analysis indicated that this enhancer has ACGT core sequences for XBP1s binding. Hence, XBP1 siRNA prevents the induction of IFN-β and XBP1s-regulated chaperone ERdj4 in macrophages.

#### 3.3.4. Eosinophils and Dendritic Cells (DCs)

XBP1 is required for eosinophil development but not for other granulocyte lineages, such as basophils and neutrophils [112]. Eosinophils are involved in type 2 immune responses, allergies, and parasitic infections [113]. XBP1s gradually increases during eosinophil differentiation, leading to the induction of XBP1s target genes, such as *P4hb*, *Edem1*, and *Sec24d* [112]. The hematopoietic deletion of XBP1 reduces the viability of eosinophil progenitors, resulting in the loss of eosinophils in the bone marrow, spleen, and blood. XBP1 ablation reduces the expression of genes responsible for maintaining ER homeostasis, thereby blocking the post-translational maturation of key granule proteins needed for survival. These defects inhibit the expression of *Gata1* that encodes a key transcription factor for eosinophil development.

Several studies have shown that XBP1 plays an important role in the development, survival, innate immune responses, and ER homeostasis of DCs [72,114,115]. DCs play a critical role in sensing pathogens and in the initiation of innate and adaptive immune responses. XBP1s is highly expressed in DCs compared with inactivated T and B cells [72]. In lymphoid chimeric mice lacking XBP1, the number and survival of conventional and plasmacytoid DCs are reduced. XBP1-deficient plasmacytoid DCs show poorly developed ER, disorganized cisternae, and decreased IFN-α production. ER stress increases the expression of IFN-β and inflammatory cytokines in polyIC-stimulated DCs [114]. However, XBP1 silencing reduces IFN-β expression, whereas XBP1s overexpression enhances the production of IFN-β, TNF-α, and IP-10 as well as DC-mediated antiviral responses. CD8α^+^ conventional DCs (cDCs) induce IRE1α activity and XBP1s expression in the absence of ER stress [115]. XBP1 loss in CD8α^+^ cDCs results in abnormal ER morphology and defective antigen presentation. Transcriptome analysis indicated that XBP1 deletion downregulates several genes responsible for protein quality control (*Rpn1*, *Rpn2*, *Edem1*, and *Stt3A*), protein folding (*Erp44* and *Dnajc10*), vesicle trafficking from ER to Golgi (*Ergic3*), and Ca^2+^ homeostasis (*Stim1* and *Stim2*). Of note, XBP1 deficiency in CD8α^+^ cDCs enhances RNase activity of IRE1α, leading to regulated IRE1α-dependent decay (RIDD). Hyperactivated IRE1α reduces the mRNA levels of *Itgb2*, *Eif2ak3*, *Ergic3*, and *Tapbp*, contributing to defects in function and antigen cross-presentation. XBP1s inhibits the antitumor immunity of DCs, thereby promoting OvCa progression [79]. OvCa increases *Xbp1* splicing in tumor-associated DCs. XBP1s induces a TG biosynthetic program in DCs, which results in abnormal lipid accumulation. However, XBP1 silencing in DCs restores antitumor immunity.

### 3.4. Cancer

Cancer cells are exposed to several stress types, including hypoxia, nutrient deprivation, and pH changes, which activate UPR by increasing unfolded proteins in the ER lumen [116]. Thus, UPR acts as a major factor in regulating tumorigenesis, progression, and metastasis. XBP1s plays a crucial role in various tumors, including triple-negative breast cancer (TNBC), melanoma, hepatocellular carcinoma (HCC), ER-positive breast cancer, and colorectal cancer [104,117,118,119,120,121]. XBP1s is increased in TNBC cells; however, XBP1 silencing inhibits tumor growth and invasiveness [117]. XBP1s upregulates the expression of the hypoxia response pathway genes by interacting with HIF1α. This cooperation increases the tumorigenicity, progression, and recurrence of TNBC by controlling the HIF1α transcriptional program. Moreover, that study identified 96 genes that were directly bound to and upregulated by XBP1s. XBP1s expression is also elevated in human melanoma tissues and cell lines [118]. The overexpression of IRE1α or XBP1s enhances IL-6 expression, thereby activating the JAK/STAT3 pathway and promoting melanoma cell proliferation. XBP1s binds to putative UPR elements containing the ACGT core sequence of the *Il6* promoter, thereby activating the transcription of *Il6* in melanoma cells. Anti-IL-6 antibodies abolish STAT3 phosphorylation and block the proliferation of melanoma cells. The critical role of XBP1s in *Il6* transcription has also been demonstrated in HCC [119]. An IRE1α RNase inhibitor, 4μ8C, blocks *Xbp1* splicing and attenuates IL-6 expression in HCC. XBP1s regulates the transcription of nuclear receptor coactivator 3 (*NCOA3*) in luminal/ER-positive breast cancer [120]. NCOA3, an oncogenic coactivator, has been implicated in the development of breast cancer [122,123]. ER stress inducers or estrogen upregulate NCOA3 expression in MCF7 and T47D cells, depending on the IRE1α–XBP1s pathway [120]. XBP1s binds to the consensus XBP1-binding site (−119 to −98) of the *NCOA3* promoter. Of note, NCOA3 induces XBP1 upon estrogen stimulation, indicating a positive feedback regulatory loop comprising XBP1s and NCOA3. The knockdown of NCOA3 inhibits the activation of the PERK–eIF2α–ATF4 pathway in response to ER stress, thereby attenuating the induction of VEGFA and LAMP3. In colorectal cancer cells, XBP1s suppresses the expression of TAp73, which is a member of the tumor suppressor p53 family [121]. TAp73 suppresses tumor cell migration, invasion, and angiogenesis [124,125]. Thapsigargin or hypoxia induce XBP1s in HCT116 cells but inhibit TAp73 expression via the binding of XBP1s to GACG sequence (−244 to −241) of its promoter [121]. Thus, XBP1s inhibits TAp73 target genes, such as apoptosis factor BIM and BAK and subsequently promotes tumor cell proliferation. All these studies indicate that XBP1s is a crucial regulator of tumorigenesis by regulating the transcription of tumor cell-related genes.

### 3.5. Alpha-1 Antitrypsin Deficiency (AATD)

AATD is a conformational disease that is associated with aberrant protein accumulation. Alpha-1 antitrypsin (AAT) is a glycoprotein that is produced primarily by the liver and secreted into the circulation [126]. AAT inhibits serine proteases, including neutrophil elastase, proteinase 3, and cathepsin G, which are secreted by neutrophils at sites of inflammation [127]. However, AATD fails to protect the lung from damage caused by the proteolytic activity of these enzymes, leading to the early onset of emphysema and chronic obstructive pulmonary disease [128]. AAT is encoded by the *SERPINA1* gene and the Z variant is the primary cause of AATD by producing mutant AAT (ZAAT) with the substitution of lysine for glutamate 342 [129,130]. ZAAT misfolds and aggregates in the ER of hepatocytes, leading to ER stress, which increases the incidence of liver damage, fibrosis, cirrhosis, and carcinogenesis [130,131,132,133]. Mutant fibrinogen also accumulates within the hepatocellular ER, leading to inherited hypofibrinogenemia [134].

All three branches of UPR are activated in response to ZAAT overexpression in several cell lines, including CHO, HEK293, HepG2, and 16HBE14o cells [135,136,137,138]. ZAAT retention in ER activates NF-κB via ER overload response (EOR) and increases expression of BiP and GRP94 via UPR [135]. Overexpression of ZAAT in HepG2 cells increases ATF6 cleavage induced by tunicamycin [136]. AAT is secreted not only by hepatocytes but also by monocytes, alveolar macrophages, and intestinal and bronchial epithelial cells [138,139,140]. Human peripheral blood monocytes from ZZ homozygous individuals showed activation of UPR, including ATF4 and XBP1s, and an increase in a subset of genes, including chaperones (*Grp58*, *Grp78*, and *Grp94*) and ERAD components (*Derlin-1* and *p97*) [139]. This contributes to an inflammatory phenotype in which NF-κB activation and production of cytokines (IL-6, IL-8, and IL-10) is enhanced. However, the roles of XBP1s in the regulation of target genes and cellular functions in AATD are still poorly understood.

Contrary to the findings above, several studies have shown that ZAAT accumulates in the polymerized form in ER and fails to induce UPR [141,142]. ZAAT also did not activate UPR even in human liver tissue, whereas nonpolymerogenic mutant AAT elicited UPR [141]. Studies using cell lines and transgenic mouse models with inducible expression of ZAAT showed that ZAAT activates specific signaling pathways, including NF-κB, caspase-4 and -12, and BAP31, and autophagy [141]. Autophagy plays a crucial role in the disposal and prevention of toxic accumulation of insoluble ZAAT [143]. Although ZAAT expression is insufficient to induce the UPR, it rendered cells hypersensitive to ER stress induced by tunicamycin or glucose depletion [144].

XBP1s expression is increased in cell lines and tissues of hepatocellular carcinoma (HCC). XBP1s contribute to the invasion and metastasis of HCC by increasing the expression of Twist and Snail [145]. For transcriptional regulation of *Twist*, XBP1s binds to the *Twist* promoter and activates promoter activity. Thus, overexpression of XBP1s promoted epithelial–mesenchymal transition (EMT) and metastasis of HCC cells, whereas XBP1s silencing attenuated cellular migration and the development of the EMT phenotype. Interestingly, HCC cells induce ER stress and activate the IRE1α–XBP1s pathway in hepatic stellate cells, thereby contributing to their activation [146]. Therefore, inhibition of IRE1α by 4μ8c in stellate cells decreases tumor burden in a mouse model of HCC. Further studies are needed to determine whether ER stress caused by the accumulation of ZAAT in AATD activates XBP1s and how increased XBP1s promotes liver injury and the development of HCC.

### 3.6. Neurodegenerative Disorders

Neurodegenerative diseases are characterized by progressive loss of neuronal function and structure [27]. Accumulation and aggregation of misfolded protein in the brain play a crucial role in the pathogenesis of many neurodegenerative diseases, such as Alzheimer’s disease (AD), Parkinson’s disease (PD), amyotrophic lateral sclerosis (ALS), Huntington’s disease (HD), prion-related disorders (PrD), retinitis pigmentosa (RP), and some myelin-related disorders [27]. Therefore, these diseases are often described as protein misfolding disorders (PMDs), which are caused by the accumulation of specific misfolded proteins: amyloid-β (Aβ) and tau in AD; α-synuclein, ubiquitin, and tau in PD; TAR DNA-binding protein 43 (TDP-43) and superoxide dismutase 1 (SOD-1) in ALS; huntingtin protein in HD; prion protein in PrD; rhodopsin in RP; and myelin in demyelinating disorders [27,147,148,149,150]. Accumulating evidence suggests that ER stress and UPR are directly involved in the physiopathology of neurodegenerative diseases.

Several studies have reported that XBP1s plays an important role in neurodegenerative disorders and brain functions [151,152,153,154]. XBP1 ablation in the nervous system results in multiple functional deficits in hippocampal synapses, leading to specific impairment of contextual memory formation and long-term potentiation (LTP) [151]. XBP1 deficiency induces decreased mRNA levels of brain-derived neurotrophic factor (*Bdnf*), a key component in memory consolidation, with moderate decreases in genes involved in neurotransmission and synaptic plasticity, including *Kif17* and *Ampa3*. XBP1s directly binds to the proximal promoter IV region of *Bdnf* gene, increasing promoter activity [151]. This binding site has a conserved sequence of UPRE B (CTCACGTCA), which is located 108 bp upstream of the transcription start site, and the ACGT core is critical for XBP1s binding. Therefore, enforced expression of XBP1s in the hippocampus improves higher cognitive functions involved in learning and memory-related processes.

In contrast to the regulatory roles of XBP1s in memory formation, activation of the IRE1–XBP1 pathway in human brain tissue is positively correlated with the progression of AD histopathology [152]. Loss of synaptic function and neuronal cell death in AD is mediated by the abnormal deposition of misfolded Aβ peptide and hyperphosphorylated tau in the brain [155]. Genetic ablation of the IRE1 RNase domain in the nervous system reduces amyloid deposition, the content of Aβ oligomers, and astrocyte activation in the cortex and hippocampus [152]. Furthermore, IRE1 deletion reduces the expression of amyloid precursor protein (APP) in cortical and hippocampal areas of AD mice. Therefore, IRE1 deficiency fully restores the learning and memory capacity of AD mice. Of note, XBP1s overexpression stabilizes APP, indicating that IRE1–XBP1s enhances APP expression and triggers AD pathogenesis. A genome-wide approach has identified a subset of XBP1s target genes that link XBP1s to neurodegenerative diseases, including AD and inclusion body myositis [156]. XBP1s regulates a transcriptional program of genes involved in APP metabolism, trafficking, and processing. XBP1s binds to the promoters of key components of the γ-secretase complex (*Ncstn*, *Psen1*, and *Psenen*) involved in APP processing. Additionally, XBP1s binds to the promoters of regulators of APP trafficking and processing (*Ubqln1*, *Apba3*, and *Apbb3*). This study also found that XBP1s binds to genes associated with the pathogenesis of AD, such as *Cdk5* and *Tcfcp2* [156]. Contrary to the roles of XBP1s in promoting AD pathogenesis, XBP1s indirectly reduces the expression and activity of β-site APP cleaving enzyme 1 (BACE1), which cleaves βAPP to produce Aβ [153]. As a mechanism for this, XBP1s increases the transcription of HRD1, which acts as a ubiquitin ligase in the ERAD process, thereby decreasing BACE1 expression.

Recent studies have shown that the mitochondria-associated ER membranes (MAMs), a site of ER in contact with mitochondria, have been implicated in a variety of neurodegenerative disorders [157,158,159,160]. The MAM integrates many signaling pathways and is important for cellular survival because it serves as the tunnel for lipid transport and Ca^2+^ signaling between ER and mitochondria [161,162]. Alterations in MAM are closely related to mitochondrial dysfunction. MAM regulates cell survival via the sigma-1 receptor (Sig-1R), an ER chaperone that specifically localizes at the MAMs [159]. IRE1 predominantly localizes at the MAM of the ER membrane. Under ER stress, IRE1 is stabilized by direct interaction with Sig-1Rs, resulting in an increase in cell survival by inducing long-lasting activation of IRE1. However, Sig-1R knockdown impairs IRE1–XBP1 signaling, leading to apoptosis. Aβ25–35 activates IRE1α–XBP1 signaling pathway and increases XBP1s expression in a human neuroblastoma cell line, SH-SY5Y cells [160]. Aβ treatment increases cytotoxicity and induces mitochondrial dysfunction, such as loss of ATP content and mitochondrial membrane potential and increase in ROS production. Aβ also increases the formation of MAMs and ER–mitochondrial interactions. Aβ increases the expression and interaction of IP3R, Grp75, and VDAC1, leading to a tethered complex on MAMs. An IRE1α inhibitor, 4μ8c, effectively mitigated Aβ-induced cytotoxicity, MAMs malfunction, and mitochondrial dysfunction by affecting MAMs.

XBP1 ablation in models of ALS and HD has protective effects through upregulation of autophagy [154,163]. Mutations in superoxide dismutase-1 (SOD1) cause ALS, whereas XBP1 deficiency in motoneurons enhances autophagy-induced clearance of mutant SOD1 aggregates, reducing its toxicity [154]. Expansion of a poly-glutamine track in Huntingtin (Htt) protein induces the accumulation of misfolded mutant Htt (mHtt), leading to HD [163]. XBP1 deficiency improves neuronal survival and motor performance and induces a decrease in mHtt levels due to enhanced autophagy [163]. This protective effect of XBP1 deficiency is mediated by increased expression of forkhead box O1 (FoxO1), a key transcription factor regulating autophagy in neurons. However, XBP1 deficiency did not modify prion-related disorders [164]. Prion aggregation, neuronal loss, and animal survival were not affected by XBP1 deficiency. Taken together, the therapeutic effects of the IRE1–XBP1 pathway in neurodegenerative disorders have different outcomes depending on the disease type. Moreover, the mechanisms by which XBP1s induces protective and regulatory effects have not yet been fully elucidated. It is necessary to identify the exact target genes of XBP1s and transcriptional regulatory mechanisms in each disease-associated cell.

## 4. Proteins Interacting with XBP1s

**Figure 4:**
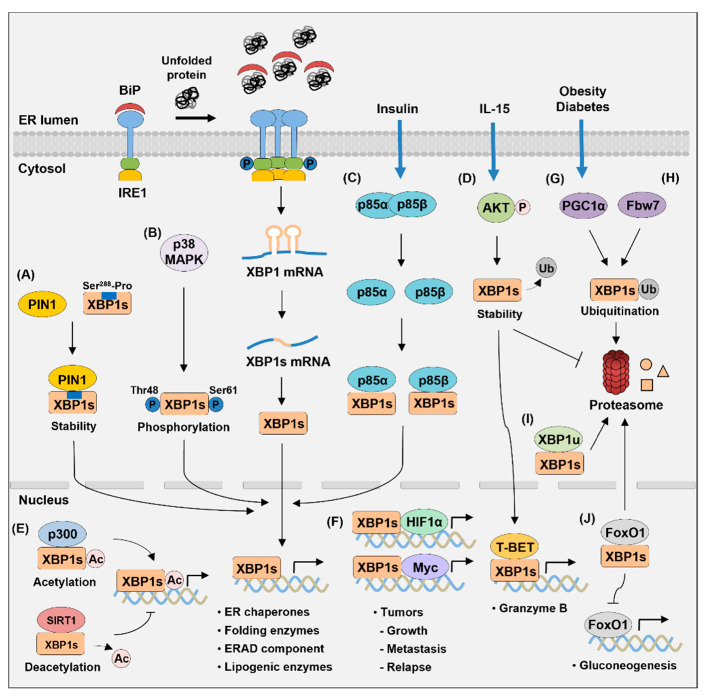
Proteins interacting with XBP1s. XBP1s interacts with a variety of other proteins, and these molecular mechanisms are important for regulating the protein stability and transcriptional activity of XBP1s. (**A**) XBP1s interacts with PIN1 in a phosphorylation-dependent manner, and this interaction increases the stability of XBP1s. XBP1s interacts with the WW domain of PIN1 via the Ser288-Pro motif of XBP1s. (**B**) The p38 MAPK directly phosphorylates the Thr48 and Ser61 residues of XBP1s, thereby enhancing the nuclear translocation of XBP1s [134]. (**C**) Insulin induces the interaction of XBP1s with p85α and p85β, the regulatory subunits of PI3K, leading to increased nuclear translocation of XBP1s. (**D**) IL-15 induces the phosphorylation of AKT, which increases the stability of XBP1s by inhibiting ubiquitination. XBP1s interacts with T-BET to bind to the proximal region of the granzyme B promoter. (**E**) XBP1s is regulated at the post-translational level via acetylation and deacetylation, mediated by its interaction with p300 and SIRT1, respectively. (**F**) XBP1s forms a transcriptional complex with HIF1α, which increases the tumorigenicity and progression of triple-negative breast cancer (TNBC). XBP1s also forms a complex with MYC, enhancing the transcriptional activity of XBP1s in tumors. (**G**) PGC1α reduces the XBP1s stability, which negatively affects the activity and protein level of XBP1s. PGC1α and XBP1s interact with each other in their activation domains to promote the protein degradation of XBP1s via ubiquitination. (**H**) Fbw7 interacts with XBP1s in a phosphorylation-dependent manner to promote the ubiquitination and degradation of XBP1s. (**I**) XBP1u functions as a negative feedback regulator of XBP1s via direct interaction. The complex of XBP1u and XBP1s is rapidly degraded by the proteasomes due to the degradation domain contained in XBP1u. (**J**) XBP1s binds directly to the FoxO1 and facilitates the degradation of FoxO1 via the 26S proteasome pathway. FoxO1 is a transcription factor that regulates gluconeogenesis by increasing the expression of phosphoenolpyruvate carboxykinase and glucose 6 phosphatase.

Recent studies indicated that XBP1s interacts with various other proteins, and these molecular mechanisms are important in regulating the protein stability and transcriptional activity of XBP1s (Figure 4). The identification of proteins that interact with XBP1s will greatly improve our understanding of the modulation of XBP1s activity. Moreover, it will help in the development of new therapies suitable for various physiological and pathological situations.

The transcriptional activity of XBP1s is regulated at the post-translational level via acetylation and deacetylation, mediated by its interaction with p300 and SIRT1, respectively [165]. p300 is a histone acetyltransferase, and SIRT1 is a class III histone deacetylase. They both regulate the acetylation/deacetylation of non-histone proteins, including transcription factors, and histone proteins [166,167]. p300 increases the acetylation and protein stability of XBP1s, but SIRT1 induces the deacetylation of XBP1s and decreases its activity [165]. XBP1s and SIRT1 colocalize in the nucleus and physically bind to each other. Thus, SIRT1 deficiency in MEF cells upregulates the expression of XBP1s target genes, including *Edem1*, *Ero1a*, and *Sec61a1*, and increases resistance to cell death under ER stress. Studies have shown that XBP1u functions as a negative feedback regulator of XBP1s via direct interaction. The expression of XBP1u increases during the recovery phase of ER stress, and XBP1u interacts with XBP1s in HeLa and auditory cells [168,169]. Proteasomes degrade this complex because of the degradation domain contained in XBP1u. Moreover, XBP1u inhibits the upregulation of inducible nitric oxide synthase (iNOS) by XBP1s during ER stress [170]. XBP1u and XBP1s form a heterodimer complex in HepG2 cells, and XBP1u overexpression decreases XBP1s protein levels.

PPARγ coactivator-1α (PGC1α), which promotes hepatic gluconeogenesis, negatively affects the activity and protein level of XBP1s by decreasing the stability of XBP1s [171]. The increased expression of PGC1α in MEF cells reduces the level of XBP1s protein. Thus, PGC1α inhibits the expression of XBP1s target genes in mouse hepatocytes. This is because PGC1α and XBP1s interact in their activation domains, promoting the protein degradation of XBP1s via ubiquitination. XBP1s also interacts with p85α and p85β, the regulatory subunits of phosphatidylinositol 3-kinase (PI3K), which increase the nuclear translocation of XBP1s [172]. p85 interacts with XBP1s through its B-cell receptor homology (BH) domain. Therefore, the expression of p85α or p85β upregulates the mRNA levels of XBP1s target genes, such as *Dnajb9*, *Pdia3*, and *Herpud1*, in MEF cells. Insulin stimulation suppresses the association between p85α and p85β; however, it promotes the binding of p85 to XPB1s and increases the translocation of XBP1s to the nucleus. The p38 mitogen-activated protein kinase (p38 MAPK) directly phosphorylates the Thr48 and Ser61 residues of XBP1s, thereby enhancing the nuclear translocation of XBP1s [173]. In the liver of obese mice, XBP1s cannot migrate to the nucleus because the interactions of p85 regulatory subunits with XBP1s are lost, and the phosphorylation of p38 MAPK is not increased [172,173]. However, expression of the constitutively active form of MAP kinase kinase 6 (MKK6) activates p38 MAPK, which increases the nuclear migration of XBP1s and reduces ER stress in obese and diabetic mice [173]. Moreover, the induction of XBP1s nuclear translocation improves glucose tolerance and reduces blood glucose levels in obese mice. XBP1s binds directly to the forkhead box O1 (FoxO1), which facilitates the degradation of FoxO1 via the 26S proteasome pathway [174]. FoxO1, a transcription factor, regulates gluconeogenesis by increasing the expression of phosphoenolpyruvate carboxykinase (*Pck*) and glucose 6 phosphatase (*G6pc*). Thus, the overexpression of XBP1s reduces the protein level of FoxO1 in the liver, thereby decreasing blood glucose levels and increasing insulin sensitivity in *ob/ob* mice. Overall, these results show that XBP1s plays an important role in regulating glucose homeostasis by its interaction with other proteins. The interaction of XBP1s with FoxO1 also occurs in auditory cells and regulates autophagy induced by ER stress [169].

Several studies have reported that UPR is activated in various tumors and that XBP1s regulates tumor initiation, progression, and growth [117,175,176]. XBP1s increases the tumorigenicity and progression of TNBC by forming a transcriptional complex with hypoxia-inducing factor 1α (HIF1α) [117]. The ChIP-seq analysis of XBP1s in MDA-MB-231 cells showed a significant enrichment of both HIF1α and XBP1s motifs, indicating that both transcription factors bind to the same regulatory elements. HIF1α interacts with the amino-terminal b-ZIP domain of XBP1s, which is critical for the expression of HIF1α target genes, including *VEGFA*, *PDK1*, *GLUT1*, and *DDIT4*. Therefore, deleting XBP1s effectively inhibits tumor growth, metastasis, and relapse in breast cancer models. A recent study has shown that XBP1s forms a transcriptional complex with MYC, enhancing the transcriptional activity of XBP1s [175]. MYC, an oncogenic transcription factor, is involved in the pathogenesis of various tumors [177]. The b-ZIP domain of XBP1s interacts with the central region and the transactivation domain of MYC [175]. MYC then increases the transcriptional activity of XBP1s by promoting XBP1s binding to the target genes. The direct association of MYC and XBP1s is found on the *SERP1*, *HSPA5*, and *PDIA3* promoters and they bind to the same genomic loci. Therefore, XBP1 silencing or IRE1 RNase activity inhibition effectively reduces the growth of MYC-overexpressing tumors. In addition, this study further demonstrated that MYC is an upstream activator of the IRE1–XBP1 pathway in breast cancer cells [175]. MYC binds to the proximal promoter and enhancer of the *Ire1* locus to activate the transcription of *Ire1*. Thus, MYC silencing induces the inhibition of *Xbp1* splicing by decreasing IRE1 expression. XBP1s also interacts with peptidyl-prolyl *cis/trans* isomerase (PPIase) NIMA-interacting 1 (PIN1) in a phosphorylation-dependent manner [176]. PIN1 binds to a specific phosphorylated Ser/Thr-Pro motif of the substrate protein and catalyzes the isomerization of the protein [178]. PIN1 is activated in human cancer and is involved in the stability of various proteins that promote tumorigenesis [179]. XBP1s interacts with the WW domain of PIN1, and the Ser^288^-Pro motif of XBP1s is important for this binding [176]. This interaction increases the stability of XBP1s; however, PIN1 deficiency inhibits XBP1s-induced cell proliferation and size increase. Fbw7 interacts with XBP1s in a phosphorylation-dependent manner to promote the ubiquitination and degradation of XBP1s [180]. Fbw7 is a substrate recognition component of the Skp1-Cullin-F-box (SCF)-type E3 ubiquitin ligase complex [181]. Fbw7 is known as a tumor suppressor protein that induces the ubiquitination and degradation of various oncoproteins [182]. The Ser^212^ and Ser^217^ sites of XBP1s are the degron motifs for Fbw7 binding; however, the substitution of these serine residues with alanine reduces the interaction with Fbw7 [180]. Fbw7 deficiency increases the expression of XBP1s by decreasing ubiquitination, thereby enhancing the tumorigenic function of XBP1s.

The expression of XBP1s increases in response to IL-15 in natural killer (NK) cells and XBP1s plays a vital role in the function and survival of NK cells [183]. IL-15 induces the phosphorylation of serine-threonine kinase protein kinase B (PKB; also known as AKT), which increases the stability of XBP1s protein by inhibiting ubiquitination. XBP1s interacts with T-BET to bind to the proximal region of the promoter of the granzyme B (*Gzmb*) gene, which increases the transcription of *Gzmb*. Therefore, the knockdown of XBP1s reduces the percentage of CD107a^+^ NK cells showing cytotoxicity against tumor cells and inhibits the IL-15-induced survival of NK cells.

## 5. Transcriptional Regulation of *Xbp1* Gene

XBP1 expression is regulated at the transcriptional level; however, information about its mechanism is not well known. ATF6 plays a major role in initiating XBP1 transcription by binding to the ERSE of the *XBP1* promoter in response to ER stress [59,65]. ERSE sequences (CCAAT-N_9_-CCACG) are present within the region from +33 to +51 of the human *XBP1* gene. ATF6 binds to the CCACG section of ERSE only when the CCAAT part of ERSE is bound to NF-Y [59].

XBP1s expression is increased in patients with osteoarthritis (OA) and is significantly induced by TNF-α and IL-1β [184]. This is because ATF6 specifically binds to the ERSE of the *XBP1* promoter in OA cartilage. The ATF6-binding site is localized at the 5′-flanking region of the h*XBP1* gene, and CCAAT-N_9_-CCACG sequences are essential for ATF6 binding. Therefore, ATF6 overexpression increases XBP1s protein level, while ATF6 siRNA decreases XBP1s expression. In addition, the knockdown of XBP1s increases ER stress-mediated apoptosis by inducing caspase cascade and the expression of proapoptotic genes in OA cartilage. Therefore, increasing *XBP1* transcription by ATF6 may be helpful in the OA treatment.

In pancreatic β-cells, hepatocyte nuclear factor 4α (HNF4α) regulates the transcription of *Xbp1* [185]. The HNF4α-binding sites are located at 1.4 and 2.6 kb upstream of the *Xbp1* transcription start site. Therefore, the knockdown or mutation of HNF4α decreases *Xbp1* mRNA levels in INS-1 and MIN6 cells. The deletion of HNF4α in mice also results in the loss of XBP1 expression in pancreatic islets, leading to altered ER structures and decreased Ca^2+^ level in the ER. That study showed that HNF4α and XBP1 are essential to maintain ER calcium homeostasis and glucose-stimulated insulin secretion in β-cells.

## 6. RIDD: Roles of RNase Activity of IRE1α

IRE1 recognizes a wide range of mRNAs as substrates besides *Xbp1* mRNA under ER stress and induces the degradation of these mRNAs, a process known as regulated IRE-dependent decay (RIDD) [186,187]. An early study with Drosophila S2 cells showed that RIDD is a mechanism that resolves ER stress by reducing protein loads entering ER by degrading mRNAs that encode ER-targeted proteins [186]. Recent studies have reported that RIDD has various physiological functions that regulate ER homeostasis, immune responses, lipid and drug metabolism, and miRNA degradation [23,187]. In mammalian cells, the target mRNAs for RIDD have a secondary structure similar to the stem-loop of the *Xbp1* mRNA and a cleavage site with the consensus sequence CUGCAG [23].

RIDD impairs proinsulin processing and insulin secretion in pancreatic β-cells [69]. XBP1-deficiency in β-cells induces the hyperactivation of IRE1α, resulting in the degradation of mRNAs encoding insulin-1 (INS1)- and proinsulin-processing enzymes (such as prohormone convertase 1 (PC1), PC2, and carboxypeptidase E (CPE)). Therefore, mice lacking XBP1 in β-cells have decreased serum insulin levels and elevated blood glucose levels. Of note, *Ins1* and *Ins2* mRNAs are degraded by IRE1α under chronic high-glucose conditions or ER stress [188,189]. This may be a protective mechanism to preserve the homeostasis of ER by inhibiting insulin entry into the ER of β-cells.

In the liver, RIDD plays a central role in lowering the plasma levels of TG and cholesterol [190]. Hyperactivated IRE1α in the XBP1-deficient liver degrades the lipid metabolism-associated mRNAs involved in lipogenesis (*Dgat2*, *Acacb*, and *Scd1*) and lipoprotein metabolism (angiopoietin-like protein 3 (*Angptl3*) and carboxylesterase 1 (*Ces1*)). ANGPTL3 inhibits lipoprotein lipase and endothelial lipase, and CES1 has TG and cholesterol-ester hydrolase activities. Therefore, the knockdown of IRE1α restores plasma lipid levels by inducing *Dgat2*, *Acacb*, *Angptl3*, and *Ces1* mRNA in XBP1-knockout mice. This study shows that XBP1 silencing in the liver effectively improves hepatic steatosis and liver damage in obese mice by inducing RIDD.

RIDD induces the cleavage of cytochrome P450 (*Cyp*) mRNA in the liver, leading to protection from acetaminophen (APAP)-induced hepatotoxicity [191]. APAP is converted to the toxic metabolite NAPQI by Cyp1a2, Cyp2e1, and Cyp3a4. Thus, IRE1α silencing in XBP1-deficient mice restores the sensitivity to APAP by increasing *Cyp1a2* and *Cyp2e1* mRNAs. IRE1α increases eIF2α phosphorylation by inducing the mRNA degradation of *Ppp1r15b*, which encodes the regulatory subunit of eIF2α phosphatase CReP [192]. The XBP1-deficient liver shows a decreased expression of CReP, resulting in the induction of eIF2α phosphorylation and the attenuation of protein synthesis. *Ppp1r15b* mRNA has a stem-loop structure containing the consensus sequences (CUGCAG) of RIDD targets.

IRE1α cleaves mRNA encoding the secretory μ (μS) heavy chain of Ig [193,194]. The *μS* mRNA has an IRE1 cleavage site with a consensus sequence and stem-loop structure. XBP1 deficiency in B cells increases IRE1α expression and RNase activity via the phosphorylation of S729 in the IRE1α kinase domain, thereby degrading *μS* mRNA and reducing IgM synthesis. This decrease in *μS* mRNA is restored by deleting the IRE1α nuclease domain or S729A mutation. Palmitate, a saturated long-chain fatty acid, activates IRE1α in invariant (*i*) NKT cells, leading to the degradation of *t-bet* and *gata-3* mRNA [195]. However, STF083010, an IRE1α-specific inhibitor, restores the mRNA levels of these transcription factors. Hence, dietary palmitate downregulates IL-4 and IFN-γ production in *i*NKT cells by promoting mRNA decay of *t-bet* and *gata-3*, which effectively inhibits arthritis.

RIDD activity contributes to cell death by increasing caspase-2 (CASP2) expression via the termination of microRNA biogenesis [196]. CASP2 is a premitochondrial protease that triggers apoptosis, and CASP2 expression is upregulated by ER stress. CASP-2 cleaves the BH3-only protein BID, translocating to mitochondria to activate BAX/BAK-dependent apoptosis [197]. The 3′-UTR of *Casp2* mRNA has binding sequences for miRNAs, including miR-17, miR-34A, miR-96, and miR-125b. IRE1α induces the degradation of precursors of anti-Casp2 miRNAs, thereby preventing proper DICER processing from producing mature miRNA forms. IRE1α increases the stability of thioredoxin-interacting protein (*Txnip*) mRNA by inducing miR-17 cleavage under ER stress [198]. Increased TXNIP expression activates the NLRP3 inflammasome in pancreatic β-cells, which induces procaspase-1 cleavage and IL-1β secretion; this promotes inflammation and programmed cell death.

Deleting XBP1 in tissues and cells such as the liver, pancreas, and B cells promotes the RIDD process by upregulating IRE1 expression and RNase activity. Therefore, distinguishing the target genes of each of XBP1 and IRE1 is an essential research topic for identifying the physiological function of RIDD. In addition, investigation of the molecular mechanisms by which XBP1 deficiency increases the expression and activity of IRE1 can contribute to the understanding of UPR signaling.

## 7. Conclusions

ER stress activates the adaptive cellular response UPR, and the three signaling branches of UPR coordinate to regulate transcriptional and translational programs to alleviate ER stress. XBP1s is a multitasking transcription factor among these signaling pathways and functions as a key mediator for the ER stress response. XBP1s increases the transcription of genes encoding molecular chaperones and ERAD components, thereby restoring ER homeostasis. Moreover, XBP1s is indispensable for proteostasis by regulating genes involved in ER expansion, protein entry into the ER, protein folding, glycosylation, and vesicular trafficking.

Many recent studies have shown that XBP1s participates in various cellular processes, such as lipid biosynthesis, glucose metabolism, autophagy, and immune responses. Hence, XBP1s is closely related to many diseases such as obesity, diabetes, cancer, and autoimmune and inflammatory diseases, and the disease-modulatory function of XBP1s has drawn attention as a critical target molecule for the development of therapeutics. The diverse functions of XBP1s indicate that XBP1s has a variety of target genes and regulates the transcription of these genes via a cell-specific mechanism. While some XBP1s target genes are conserved in different tissues and cells, some are unique to a given cell. Although several cis-acting elements for XBP1s binding have been elucidated, the detailed transcriptional mechanisms for regulating each gene are poorly understood.

Little information is available about the exact target gene of XBP1s for each cell and disease; however, recent transcriptional profiling analysis has facilitated the identification of XBP1s target genes in different organs, tissues, and cells. Functional classification via network analysis among global target genes helps us to understand the physiological function of XBP1s. The transcriptional activity of XBP1s is regulated through its interaction with other proteins, but the detailed mechanism for this is still largely unknown. These interactions lead to the modification of XBP1s, increased nuclear translocation and protein stability, or proteasomal degradation. XBP1s also induces the transcription of unique subsets of genes in specific cells through interactions with other transcription factors.

The elucidation of several topics is needed to understand the mechanisms of transcriptional regulation by XBP1s: (1) the target genes of XBP1s in each cell, (2) additional *cis*-acting elements for XBP1s binding, (3) binding sites of XBP1s other than the promoter, (4) a regulatory mechanism of XBP1 activity, (5) binding partners of XBP1s and its roles, and (6) the relationship between XBP1s and epigenetic regulation. Understanding the function of the XBP1s-dependent transcriptional program in each cell and disease may provide important clues for developing novel therapeutic targets for diseases. The screening of compounds targeting XBP1s is a promising strategy and potential approach for managing metabolic or inflammatory disorders.

## Data Availability

Not applicable.
